# Editorial: Advancements in cycling performance enhancement strategies for cyclists: from amateurs to elite

**DOI:** 10.3389/fspor.2025.1549760

**Published:** 2025-03-10

**Authors:** Alejandra Polanco, Paul William Macdermid, Milaim Berisha

**Affiliations:** ^1^ Laboratoire de Biomécanique et Mécanique des Chocs (LBMC), Université Gustave Eiffel, Bron, France; ^2^ School of Sport, Exercise and Nutrition, College of Health, Massey University, Wellington, New Zealand; ^3^ Sport Science and Movement Faculty, University for Business and Technology (UBT), Prishtine, Kosovo

**Keywords:** cycling, performance, editorial, competitive cycling, recreative cycling

**Editorial on the Research Topic**
Advancements in cycling performance enhancement strategies for cyclists: from amateurs to elite

The world of cycling is experiencing a transformative era, where cutting-edge technologies, innovative methodologies, and evidence-based practices are reshaping performance enhancement strategies for both competitive athletes and recreational riders, including those who use bicycles as a mode of transportation. From amateur enthusiasts to elite competitors, these advancements not only drive improvements in efficiency and endurance but also address critical challenges related to athlete well-being, recovery, and resilience. Aligned with the aim and scope of the special issue Advancements in Cycling Performance Enhancement Strategies for Cyclists: From Amateurs to Elite, the key content and benefits of the issue are outlined below (1):

• Cycling Performance: Tackling Challenges with Technology
• Innovative Methodologies in Cycling Performance
• Practical Tips for Athletes and Coaches
• The Holistic Impact on Cycling Performance

## Cycling performance: tackling challenges with technology

Self-tracking tools, including wearables and performance monitoring apps, have become indispensable for athletes aiming to optimize their performance. However, a study by Werner and Bischof reveals that these technologies introduce notable stress factors for amateur triathletes. The researchers identified 16 stressors, eight adapted from workplace contexts-such as information overload and loss of control—and eight sport-specific stressors, including measurement fixation, comparison pressure, and the performance enhancement imperative. These findings underline the dual impact of self-tracking technologies, which simultaneously offer benefits and challenges. They advocate for adaptive training programs and personalized feedback systems to minimize the adverse effects of technostress while maintaining the utility of these tools.

On a similar note, the validity of performance software is pivotal in optimizing training strategies. Poffé et al. evaluated the INSCYD software, which calculates the maximal lactate steady state (MLSS), a critical metric for defining training zones and predicting race outcomes. Their research confirmed the software's accuracy in comparison with laboratory methods, with only minor discrepancies in power output measurements. This validation positions INSCYD as an invaluable resource for athletes and coaches, streamlining evaluations and making advanced physiological insights more accessible (2).

## Innovative methodologies in cycling performance

Advanced research methodologies have enabled a deeper understanding of the multifaceted challenges in cycling. Fallon and Heron proposed a systematic review protocol to analyze injuries and illnesses across competitive cycling disciplines, including under-researched areas like BMX freestyle and para-cycling. By standardizing methodologies and definitions, their study seeks to resolve discrepancies in epidemiological data and align with the Union Cycliste Internationale's Agenda 2030. This initiative promises to provide a comprehensive overview of injury prevalence and incidence, informing policies and practices to enhance athlete safety.

Carlsson et al. investigation into physiological responses during steep uphill cycling employed a unique methodological approach to assess the effects of different riding positions. The study compared seated, standing, and alternating positions, with results showing that repeated transitions between positions every 10 s reduced blood-lactate concentrations without increasing oxygen consumption. This dynamic strategy offers a practical means to enhance efficiency and performance during high-intensity climbs, presenting a valuable addition to training regimens for elite cyclists (3).

## Practical tips for athletes and coaches

Practical insights extend beyond innovative technologies and methodologies to encompass critical aspects of nutrition and recovery (5). Peeters et al. conducted a cross-sectional survey examining indoor cycling practices. Their findings revealed that external factors, such as weather and time efficiency, drive participation in indoor cycling, with athletes predominantly using smart trainers and gamified software like Zwift. While hydration practices remained consistent, the study highlighted variations in carbohydrate and protein intake based on workout duration. These insights emphasize the importance of structured nutritional strategies tailored to specific training contexts, offering a foundation for optimized performance.

Recovery, a cornerstone of athletic success, also benefits from focused research. Javaloyes et al. explored sleep quality among elite and junior cyclists using the Pittsburgh Sleep Quality Index, revealing that 41% of athletes experienced poor sleep. Variations were observed based on gender and discipline, with female cyclists reporting lower sleep quality and endurance cyclists spending more time in bed compared to sprinters. These findings underscore the need for targeted interventions to address sleep-related challenges, highlighting the interconnected roles of recovery, physical performance, and mental well-being.

## The holistic impact on cycling performance

Together, these studies illustrate a comprehensive approach to addressing the complexities of performance-oriented cycling and triathlon. Self-tracking technologies, while indispensable, require careful integration to avoid technostress and enhance usability. Adaptive solutions such as personalized feedback and validated performance software like INSCYD empower athletes with actionable data while minimizing potential drawbacks (6).

The application of standardized research methodologies, as demonstrated by Fallon and Heron, advances the field by providing robust data on injury prevalence and risk factors. These findings are critical for shaping policies that prioritize athlete safety and long-term participation. Additionally, Carlsson et al. insights into dynamic climbing strategies introduce practical techniques that can be incorporated into elite training programs, improving performance during grueling uphill sections.

Nutrition and recovery remain vital components of an athlete's regimen. Structured approaches to carbohydrate and protein intake, as outlined by Peeters et al., ensure that athletes maximize their training outcomes. Addressing sleep challenges reinforces the importance of recovery, laying the groundwork for sustainable performance improvements (5).

These findings collectively underscore the importance of integrating technological innovation with athlete-centric strategies to enhance both achievement and well-being. By addressing challenges such as technostress, injury risks, and recovery gaps, the studies provide a roadmap for optimizing training, competition, and overall athletic experience (4). For athletes, coaches, and policymakers, these insights offer practical tools and strategies to navigate the evolving landscape of performance-oriented cycling and triathlon, ensuring a balance between technological advancement and holistic athlete welfare.
